# Supplementary material for: Influence of Crosslinking Methods on Biomimetically Mineralized Collagen Matrices for Bone-like Biomaterials
Source: Polymers (Basel). 2023 Apr 22;15(9):1981. doi: 10.3390/polym15091981 (PMC10180878; doi:10.3390/polym15091981)
Supplement: Supplementary file 1 [file polymers-15-01981-s001.zip › polymers-2301899-supplementary.pdf]

Supplementary Materials: Influence of Crosslinking Methods on Biomimetically Mineralized Collagen Matrices for Bone-like Biomaterials

Table S1. EDS mineral content measurements for mineralized collagen scaffolds. Ratios represent crosslinker to collagen mol/mol ratios.

Crosslinking Type	Crosslinking Conditions	wt% Ca	At% Ca	At% P	At% C	Ca/P Ratio	Ca/C Ratio
Quercetin	0:1	26.7 ± 0.6	12.00 ± 0.35	7.83 ± 0.08	51.40 ± 0.51	1.53	0.23
	0.5:1	27.4 ± 0.2	12.36 ± 0.11	7.93 ± 0.14	51.98 ± 0.10	1.56	0.24
	1:1	28.9 ± 1.4	13.24 ± 0.78	8.41 ± 0.39	52.37 ± 1.71	1.57	0.25
	2:1	28.0 ± 0.8	12.62 ± 36	8.08 ± 0.18	54.86 ± 2.15	1.56	0.23
Methacrylated	0s	28.6 ± 0.5	13.11 ± 0.32	8.53 ± 0.08	51.56 ± 0.65	1.54	0.25
	45s	26.7 ± 0.3	12.09 ± 0.13	7.99 ± 0.15	49.46 ± 0.61	1.51	0.24
	90s	27.8 ± 0.4	12.66 ± 0.21	8.24 ± 0.35	50.25 ± 1.21	1.54	0.25
	300s	28.2 ± 0.8	13.00 ± 0.44	8.55 ± 0.18	48.95 ± 0.33	1.52	0.27
EDC	0:1	29.7 ± 0.2	13.97 ± 0.12	8.78 ± 0.03	45.19 ± 0.54	1.59	0.31
	0.5:1	29.3 ± 0.9	13.77 ± 0.60	8.86 ± 0.35	45.82 ± 0.33	1.55	0.30
	1:1	28.1 ± 0.1	12.96 ± 0.08	8.51 ± 0.05	47.76 ± 0.33	1.52	0.27
	2:1	28.2 ± 0.3	12.95 ± 0.25	8.53 ± 0.21	49.16 ± 1.00	1.52	0.26
Riboflavin	0s	26.9 ± 0.5	11.97 ± 0.27	7.69 ± 0.19	54.56 ± 2.29	1.56	0.22
	60s	25.1 ± 0.9	11.00 ± 0.47	7.24 ± 0.29	54.87 ± 1.00	1.52	0.20
	300s	29.7 ± 4.1	13.65 ± 2.32	8.48 ± 0.67	55.30 ± 4.18	1.61	0.25

Table S2. Mechanical testing data for mineralized collagen scaffolds. The first condition for each crosslinker is the negative control, without crosslinking.

Crosslinking Type	Crosslinking Conditions	Tensile Modulus (MPa)	Ultimate Tensile Strength (MPa)
Quercetin	0:1 w/w	597 ± 84	3.4 ± 0.56
	0.5:1	838 ± 73	2.4 ± 0.31
	1:1	869 ± 64	3.8 ± 0.25
	2:1	765 ± 176	1.6 ± 0.62
Methacrylated	0 s	765 ± 246	0.7 ± 0.54
	45 s	636 ± 332	1.5 ± 0.40
	90 s	777 ± 80	2.1 ± 0.49
	300 s	387 ± 47	1.8 ± 0.57
EDC	0:1 w/w	2586 ± 591	2.6 ± 2.10
	0.5:1	2146 ± 239	28.8 ± 6.60
	1:1	1255 ± 424	43.2 ± 9.70
	2:1	561 ± 261	67.4 ± 10.0
Riboflavin	0 s	4743 ± 1456	4.0 ± 0.31
	60 s	3000 ± 662	18.7 ± 6.78
	300 s	230 ± 84	12.2 ± 3.38

Table S3. Mechanical testing data for non-mineralized collagen scaffolds.

Crosslinker	Crosslinking Conditions	Tensile Modulus (MPa)	Ultimate Tensile Strength (MPa)
Quercetin	2:1*	308 ± 29	45.8 ± 4.3
Methacrylated	300 s*	51 ± 5	7.5 ± 1.6
EDC	0:1 w/w	230 ± 14	38.4 ± 3.3
	0.5:1	492 ± 105	65.2 ± 3.5
	1:1	831 ± 36	102 ± 14.4
	2:1	719 ± 94	43.75 ± 4.5
Riboflavin	0 s	230 ± 14	38.4 ± 3.2
	60 s	418 ± 10	60.8 ± 6.0
	300 s	318 ± 9	49.5 ± 6.9

* Quercetin and Methacrylated collagen did not yield reportable results for the lower degrees of crosslinking due to early failure

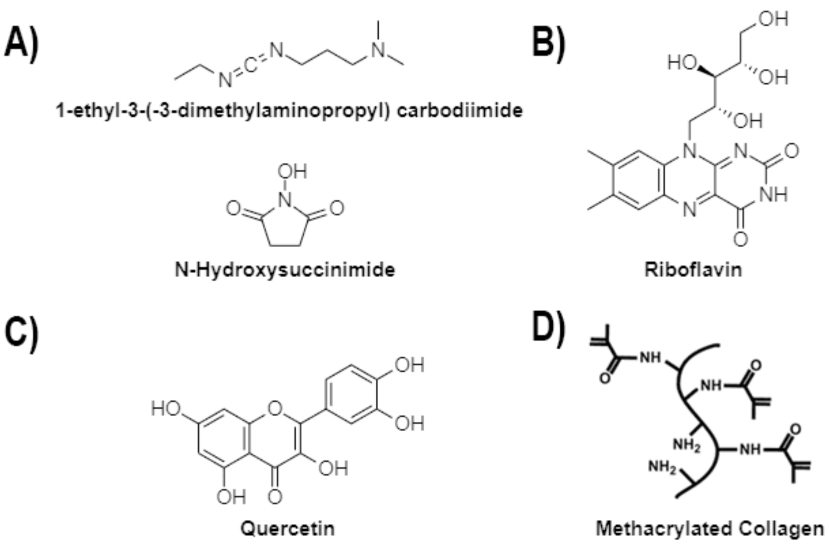

Figure S1. Structures for the crosslinkers utilized in (**A**) EDC-NHS, (**B**) Riboflavin (**C**) Quercetin crosslinking and (**D**) methacrylated collagen. Methacrylation in (**D**) is reprinted from [49] with permission from Elsevier.

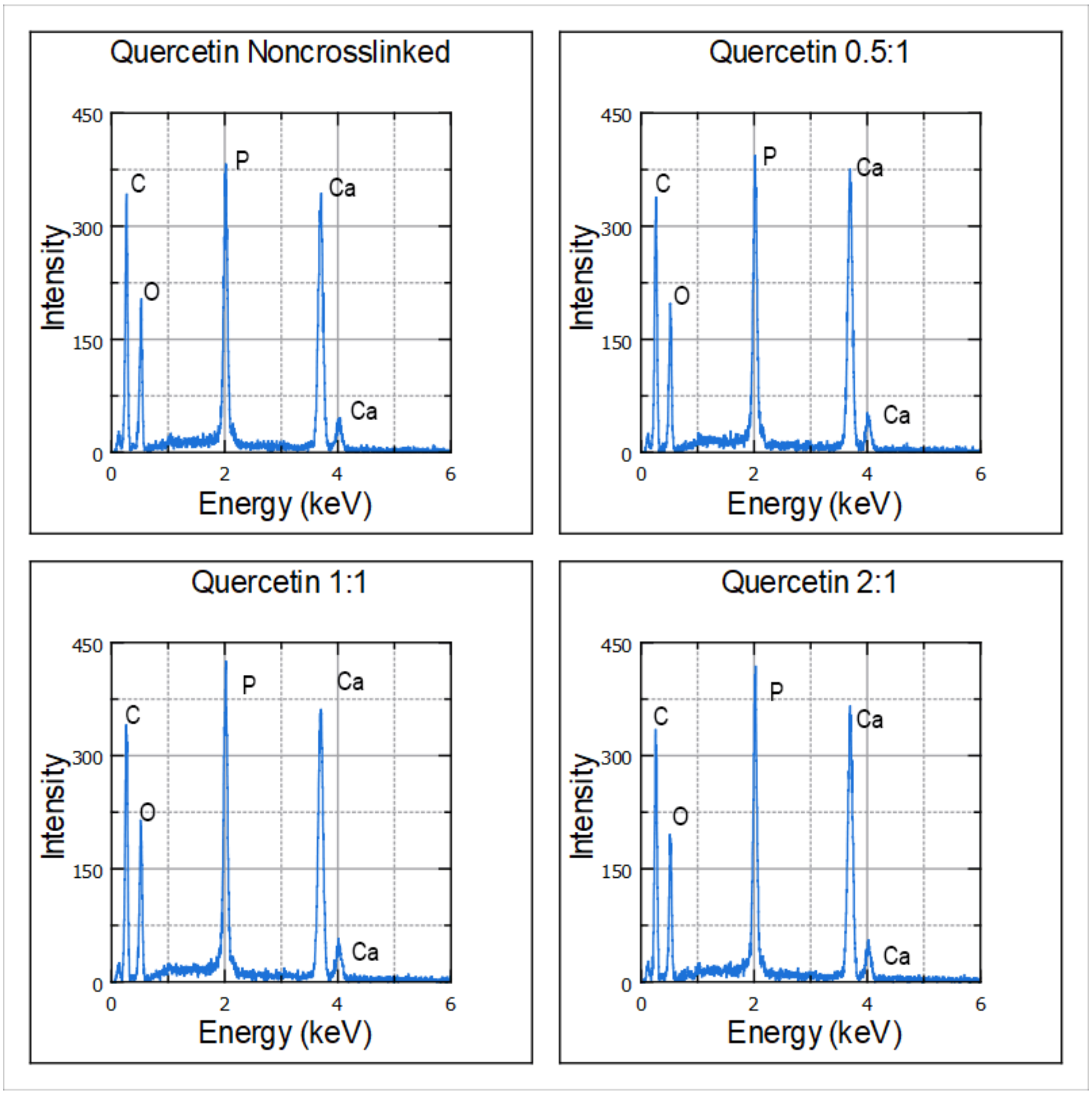

Figure S2. EDS Spectra of mineralized and Quercetin crosslinked collagen samples.

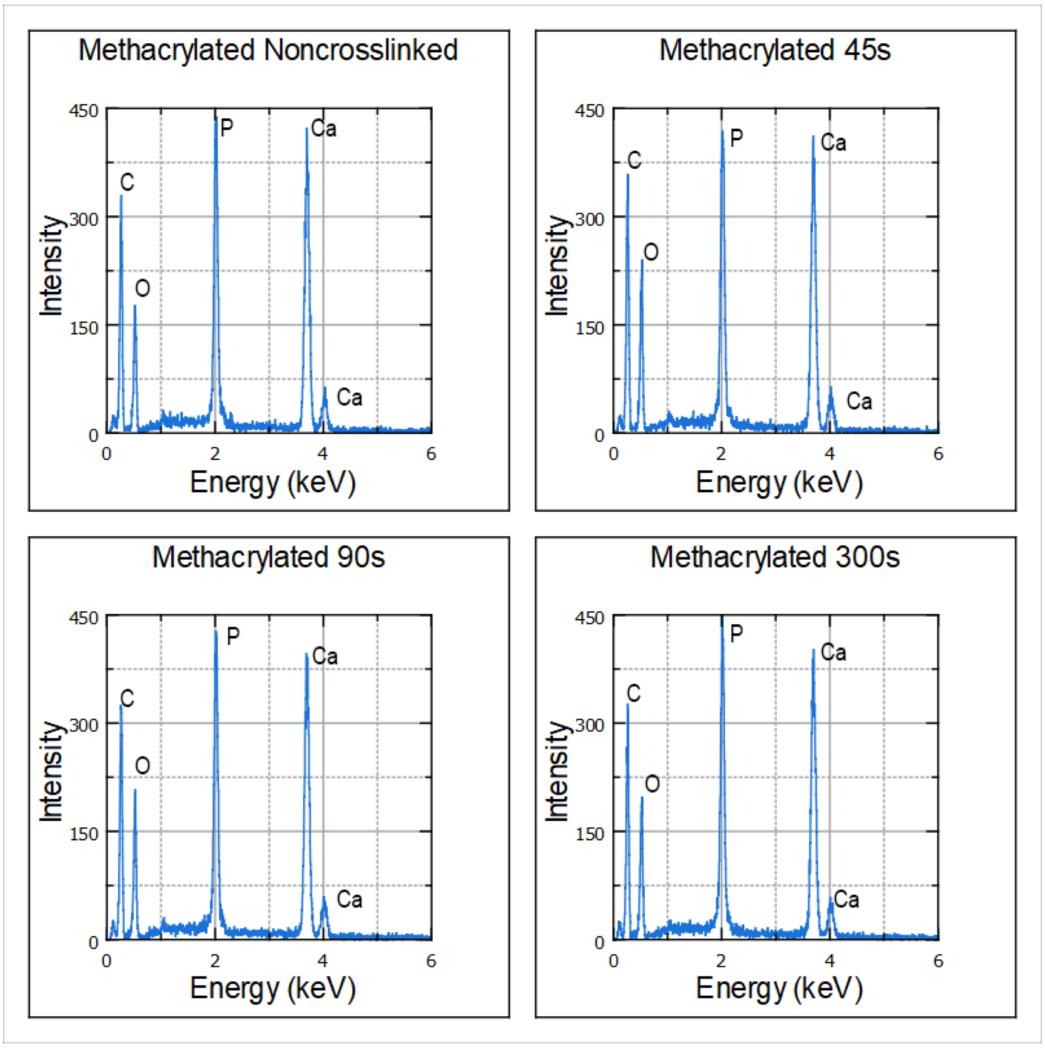

Figure S3. EDS Spectra of mineralized Methacrylated collagen samples.

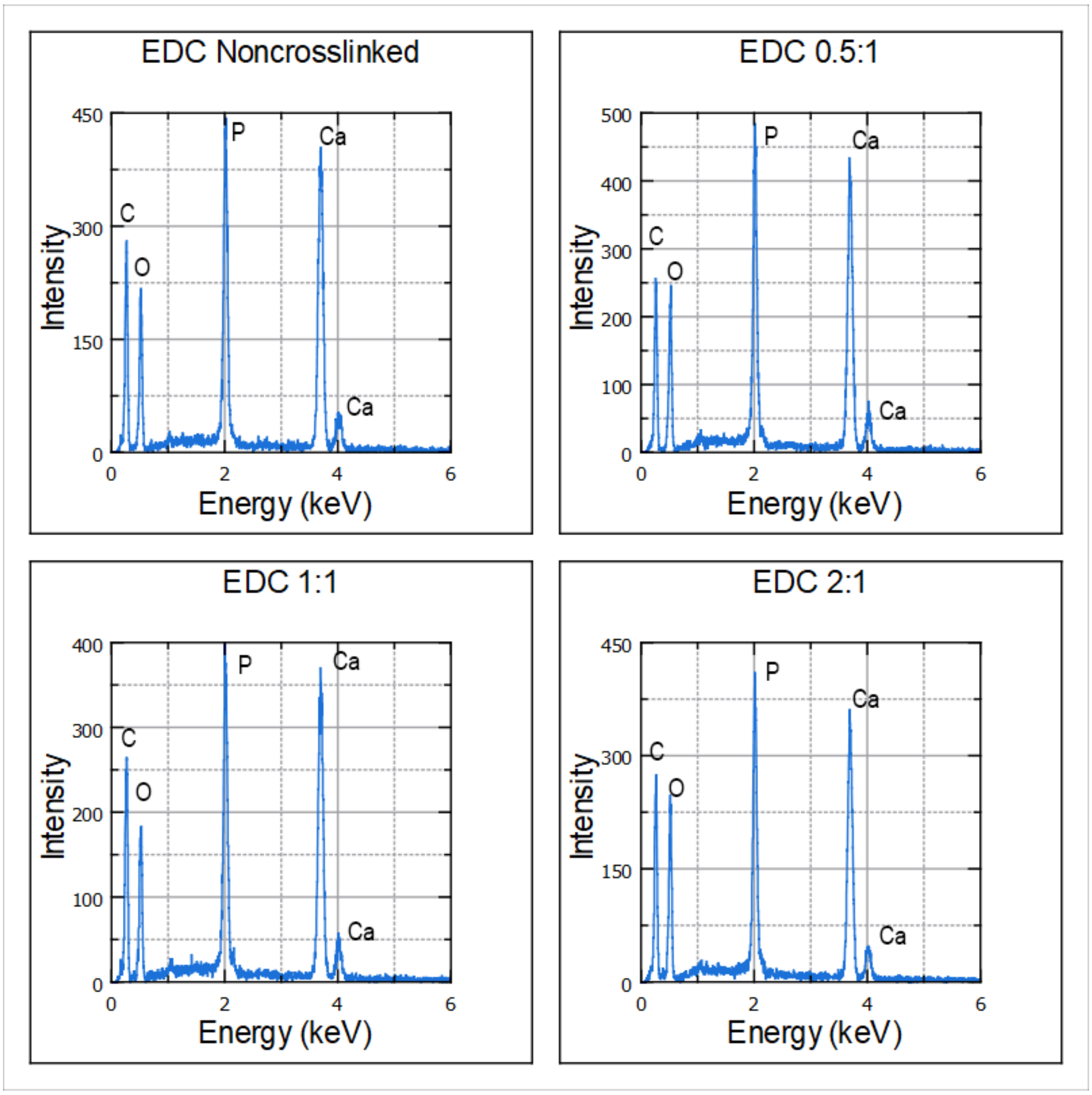

Figure S4. EDS Spectra of mineralized and EDC-NHS crosslinked collagen samples.

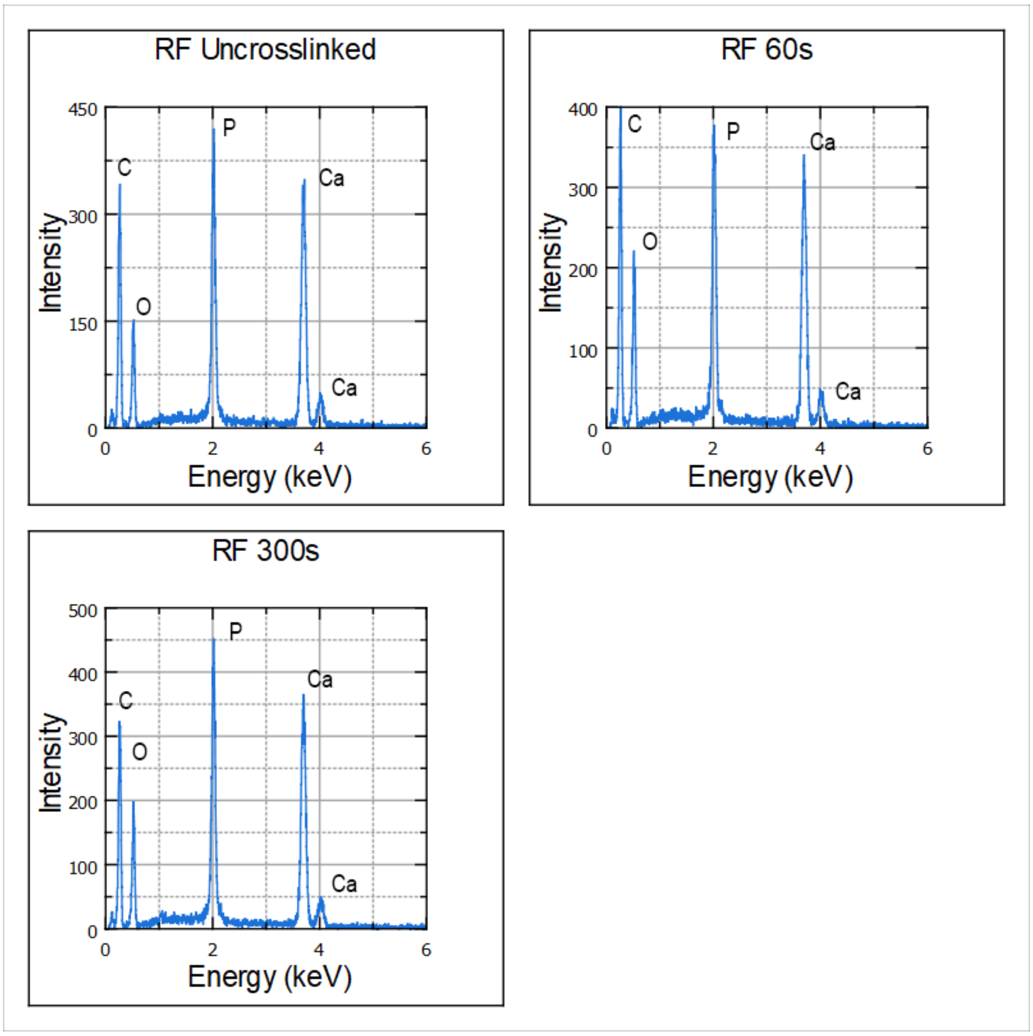

Figure S5. EDS Spectra of mineralized and Riboflavin-incorporated collagen samples.

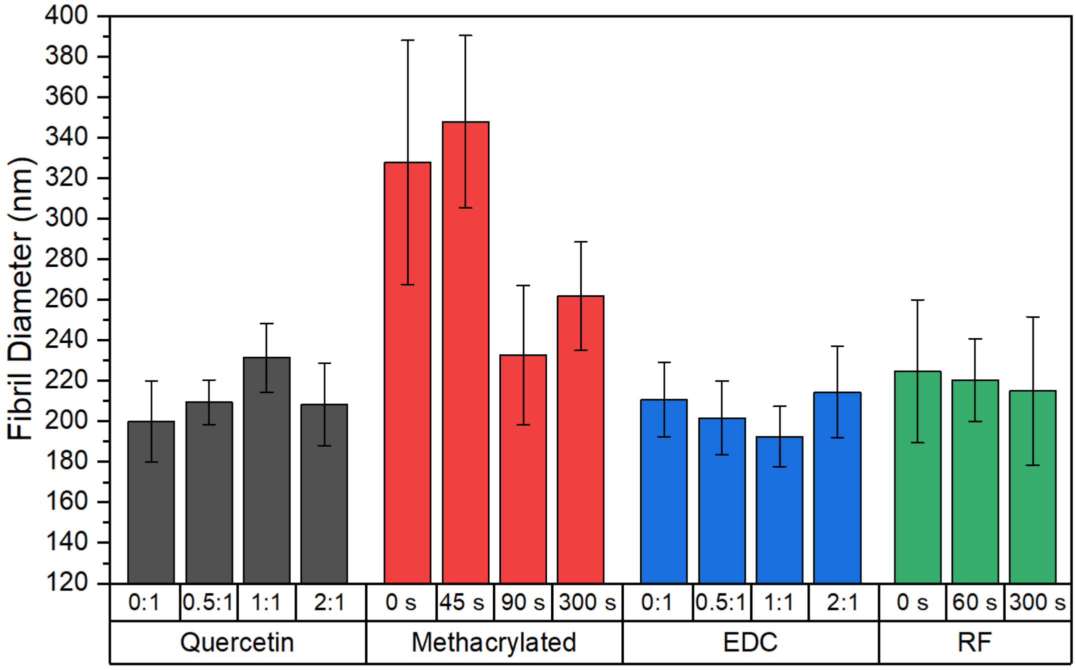

Figure S6. Fibril diameter measurements for each respective cross-linking group based on measurements of SEM images. EDC, RF and Quercetin crosslinking groups all show values in a similar

range, while values for methacrylated collagen are much higher for the first two groups. Error bars represent standard deviation.

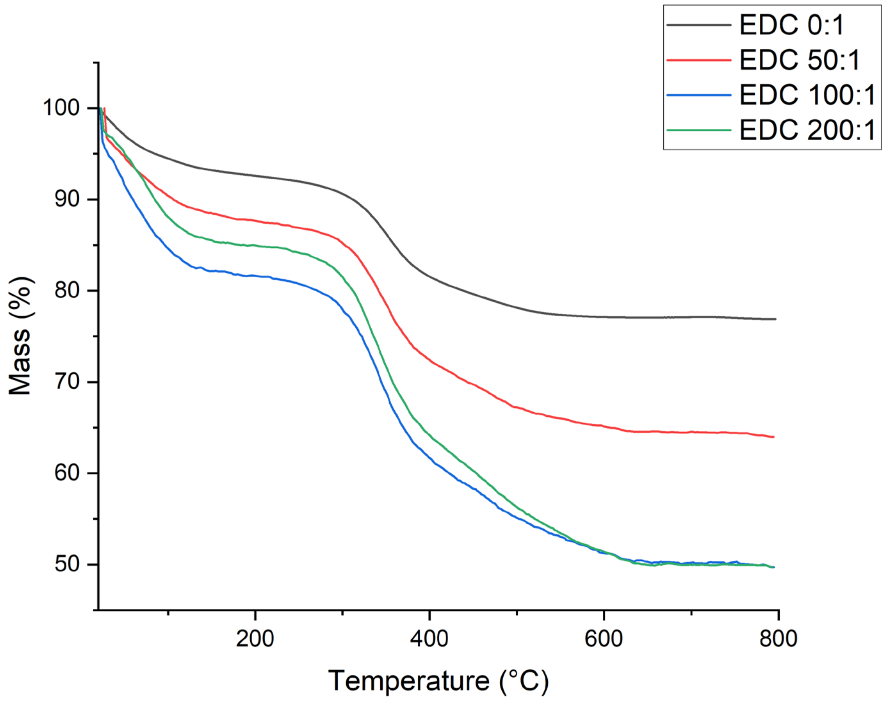

Figure S7. Thermogravimetric analysis curves for EDC-NHS crosslinked collagen.

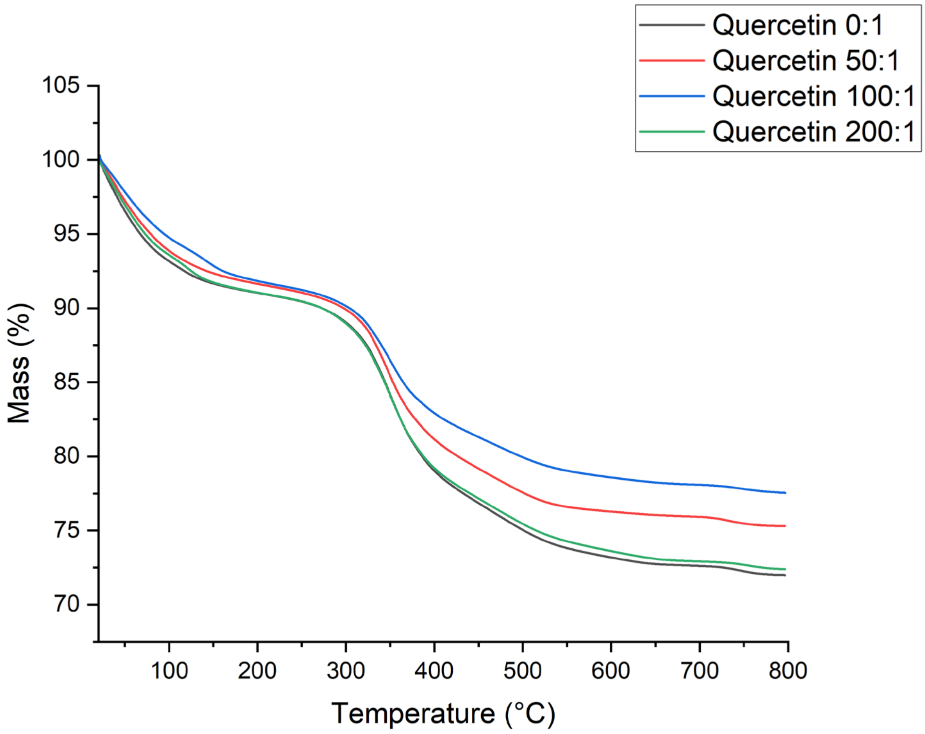

Figure S8. Thermogravimetric analysis curves for quercetin crosslinked collagen.

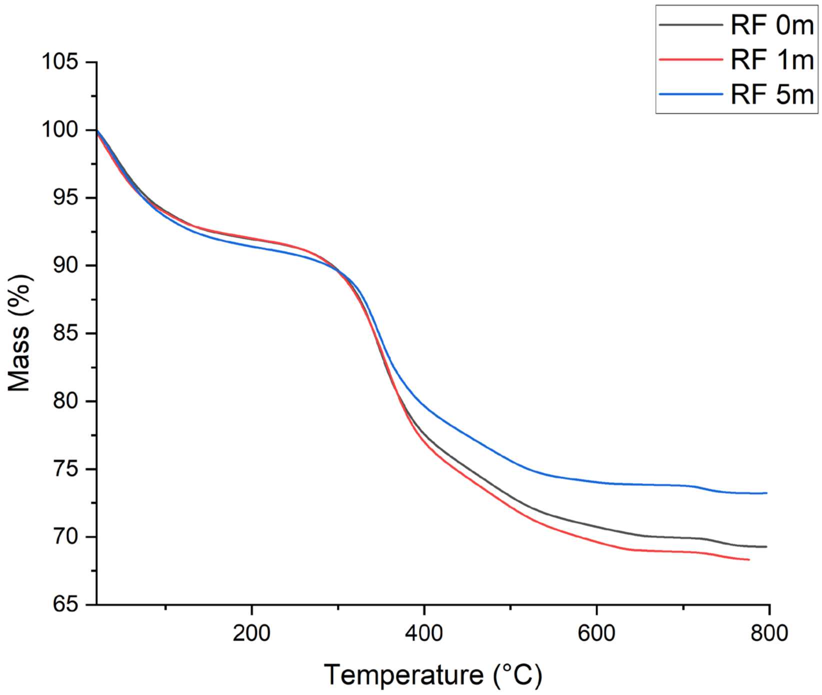

Figure S9. Thermogravimetric analysis curves for riboflavin crosslinked collagen.

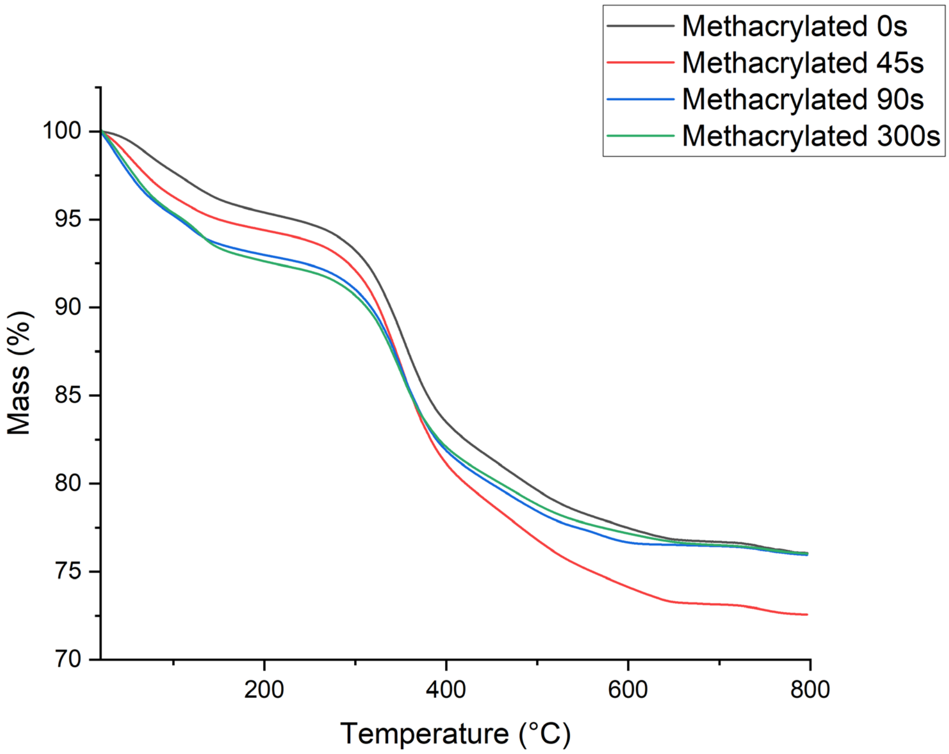

Figure S10. Thermogravimetric analysis curves for methacrylated collagen.
